# The use of NADH anisotropy to investigate mitochondrial cristae alignment

**DOI:** 10.1038/s41598-024-55780-5

**Published:** 2024-03-12

**Authors:** Holly. E. Smith, Alasdair M. Mackenzie, Chloe Seddon, Rhys Mould, Ifi Kalampouka, Partha Malakar, Sarah R. Needham, Konstantinos Beis, Jimmy D. Bell, Alistair Nunn, Stanley W. Botchway

**Affiliations:** 1 UKRI, STFC, Central Laser Facility, Rutherford Appleton Laboratory, Oxfordshire, OX11 0QX UK; 2 Department of Life Sciences, Imperial College London, London, SW7 2AZ UK; 3 Rutherford Appleton Laboratory, Research Complex at Harwell, Didcot, Oxfordshire, OX11 0FA UK; 4 School of Life Sciences, Research Centre for Optimal Health, University of Westminster, London, W1W 6UW UK

**Keywords:** Cell biology, Optics and photonics

## Abstract

Life may be expressed as the flow of electrons, protons, and other ions, resulting in large potential difference. It is also highly photo-sensitive, as a large proportion of the redox capable molecules it relies on are chromophoric. It is thus suggestive that a key organelle in eukaryotes, the mitochondrion, constantly adapt their morphology as part of the homeostatic process. Studying unstained in vivo nano-scale structure in live cells is technically very challenging. One option is to study a central electron carrier in metabolism, reduced nicotinamide adenine dinucleotide (NADH), which is fluorescent and mostly located within mitochondria. Using one and two-photon absorption (340–360 nm and 730 nm, respectively), fluorescence lifetime imaging and anisotropy spectroscopy of NADH in solution and in live cells, we show that mitochondria do indeed appear to be aligned and exhibit high anisotropy (asymmetric directionality). Aqueous solution of NADH showed an anisotropy of ~ 0.20 compared to fluorescein or coumarin of < 0.1 and 0.04 in water respectively and as expected for small organic molecules. The anisotropy of NADH also increased further to 0.30 in the presence of proteins and 0.42 in glycerol (restricted environment) following two-photon excitation, suggesting more ordered structures. Two-photon NADH fluorescence imaging of Michigan Cancer Foundation-7 (MCF7) also showed strong anisotropy of 0.25 to 0.45. NADH has a quantum yield of fluorescence of 2% compared to more than 40% for photoionisation (electron generation), when exposed to light at 360 nm and below. The consequence of such highly ordered and directional NADH patterns with respect to electron ejection upon ultra-violet (UV) excitation could be very informative—especially in relation to ascertaining the extent of quantum effects in biology, including electron and photonic cascade, communication and modulation of effects such as spin and tunnelling.

## Introduction

“Life is nothing but an electron looking for a place to rest” to quote Albert Szent-Györgyi, the Hungarian physiologist, Nobel Prize in 1937. Life is fundamentally a self-organising, replicating, far from equilibrium dissipating process^1^. An echo of this is seen in all life as the electrochemical gradient, which is especially large in mitochondrion and coupled to the merry-go-round of the Kreb’s cycle—in a way, the mitochondrion represents the ultimate “flux capacitor”^2^.

The possibility exists that the “shape” of life is determined by a bioelectric field, which could interact with photons^1^. This concept of “field homeostasis” is further suggested by the ability of biology to detect magnetic fields and be manipulated by them^3,4^, hence the flow and role of photo-electrons could be significant. This suggests that biology, to some degree, may be reliant on an underappreciated level of photonic/electromagnetic field (EMF)-based structural organisation and homeostasis in which mitochondria may play a significant role. This is perhaps further emphasised by the emerging discipline of studying the role of spintronics and the origins of chirality in biology^5^. In effect, there could be a much tighter interaction between structure, spin, and light. Life has also evolved to use light in ways that is not yet fully understood. For example, live mammalian and plant cells generate intrinsic photons, described as biophotons, ultraweak photon emission and metabolic photon emission^6,7^. These are thought to originate from metabolic process including significant proportion from the mitochondria, lipid peroxidation and radical reactions. The wavelength of these photons range from ultraviolet-A (UVA) to near infrared (NIR). It is highly likely that some of these photons would be reabsorbed by cellular components including mitochondria. Fortunately, there is one molecule that is relatively easy to study by light, which might give us some indication of this, and that is nicotinamide adenine dinucleotide (NAD). It exists as an oxidized and reduced form, abbreviated as NAD+ and NADH (H for hydrogen), respectively. NADH, which is primarily found in the mitochondrion is seen as being largely key in redox reactions in biochemistry, although it could be argued that it may also act as a sunscreen^8^.

NADH is a key electron (and proton) carrier, and although there is still debate about the metabolism versus genetics origins of life, it is fairly well accepted that life arose due to the laws of thermodynamics and entropy as a far from equilibrium self-organising dissipating structure, thus making this molecule a pivotal component of this process. Although NADH is thought of being predominantly involved in energy metabolism, in antioxidant metabolism, together with NAD+, they both function in virtually every aspect of life, such as signalling^9^. Critically, NAD(P)H is the archetypal chromophoric cofactor and is also fluorescent, which enables not just imaging of mitochondria, but also provides information on both redox and binding via changes in its lifetime decay^10^.

What is well known is that mitochondria can form reticular networks and constantly fuse and fission as part of a mechanism to adapt to metabolic conditions, including stress, infection, and the cell’s shape, which also ensures damaged ones can be removed by mitophagy. What is perhaps less well known is that these reticular networks can also transmit energy and information by way of inter-mitochondrial junctions (IMJ)^11^. Interestingly, it seems these IMJs are associated with cristae alignment between mitochondria and likely reflect their ancestral origins as a once free living, but cooperative bacterium^12^. In effect, the constantly adaptive mitochondrial dynamics seen in modern cells, not only reflects their free-living ancestry as cooperative bacteria, but also likely mirrors the adaptive changes seen in their inner membranes. This builds on the emerging evidence that different species of prokaryotes, including between bacteria and Archaea, cooperate in highly organised consortia by sharing electrons^13^.

One of the key questions, which has long been asked, is how dependent is biology on quantum mechanics—and how do we explore/measure the extent? In one respect, the first question is clearly a non-question, as it has to be when viewed at the level of single electrons and photons, but can we show that biology has utilised quantum principles that might not be expected at the temperatures it operates at? Evidence is, it has, ranging from exciton transfer in photosynthesis, to bird navigation and control of reactive oxygen species, ROS, involving electron spin, to vibrational theories to explain olfaction, and the role of tunnelling in both enzyme function and electron transport^14^. In fact, it is entirely possible that the apparently disordered environment can actually enhance quantum transport^15^. This does suggest that the mitochondrion, because of its electrical properties, is a good place to look for quantum features^16^. One potentially fruitful area of investigation therefore arises from the role of spin and polarisation, and molecules that might, potentially be a read out of quantum-based directionality, such as NAD(P)H—which might also further indicate that life also relies on vectored fields for information and signalling, and thus, homeostasis.

Anisotropy measurements provides information on the lack of isotropic nature of an object (that is, different along different directions). Generally anisotropy is mostly observed in single crystals where the atoms, ions, or molecules are in a non-regular lattices. Measurements along a particular axis leads to different values depending on whether the measurement is parallel or perpendicular to that axis. Polarised light traversing a crystal will therefore yield its symmetry information. The use of polarisation studies such as fluorescence anisotropy can provide information about the orientation, environment (including viscosity), size and dynamics (rotational freedom) of molecules. In general, light electromagnetic field oscillations can be characterised by their electric and magnetic components, which is perpendicular to the light propagation (here we only consider the electric field). Upon absorption of polarised light (dipole dependent), a molecule may emit a fluorescence photon, the direction of the electric vector (oscillates in only one direction) can be observed and determined through a polariser that is orientated in a particular direction. Generally, this is defined as parallel (∥, along the excitation diploe direction) or perpendicular (⊥, orthogonal) direction relative to the z-axis (or laboratory vertical, V, orientation). Anisotropy can therefore be used to describe polarisation^17^. In biophysics, fluorescence anisotropy studies can be used to determine molecular physical properties, including spin characteristics, rotation, molecular orientation, aggregation and energy transfer between identical molecules^18–20^. Freely rotating molecules will have very little (close to zero) anisotropy whilst those in a fixed orientation with their dipole aligned to the light electric field vector will exhibit a strong anisotropy (Fig. S1). Anisotropy (and polarisation) measurements have been used as screening tools to determine molecular binding process, for example, drug binding to proteins^21–23^. It is argued that a small molecule that is freely rotating under Brownian motion would have a low anisotropy value (as depicted in Fig. S1), upon binding to a larger molecule such as a protein, its rotation would slow leading to an increase in its anisotropy value. Similarly, since a single protein will have rotation characteristics that is different to when bound to another (protein–protein interactions or dimers), the anisotropy values for the single protein compared the dimer or complex will lead to difference in the anisotropy values determined. Generally, a dimeric protein will exhibit a lower value of anisotropy compared to a monomer or the protein alone without the complex.

The studies we describe present a novel avenue for exploring a possible fixed and ordered nature of NADH molecule particularly in mitochondria towards the beginning of understanding the other properties of the mitochondria, as well as the potential involvement of NADH in quantum biology. We envisage anisotropy and polarisation to have a significant role in such studies^24–27^. Although it is worth noting that NADH is present throughout the cell including the nucleus, it is mostly located in the mitochondria , which is why we have focussed on this organelle, as its spatio-temporal shifts can also be easily followed.

Upon light absorption a small fraction of NADH (2%) results in fluorescence emission with the majority (~ 44%) resulting in photoionisation to generate hydrated electrons in solution^28^. The photoionisation of NADH at around 360 nm (3.44 eV) is thought to be a largely one-photon process, although there is still some debate as to whether a two-photon process may contribute^25,26,29,30^. To date, the majority of NADH biophysical studies have centred on the fluorescence of NADH with only a few literature reports of the electron yield from photon absorption of NADH. Considering the high quantum yield of photoionisation, and the importance of electrons in the electron transport chain (ETC) and life itself, this is surprising.

Fluorescence polarisation or anisotropy is a technique that can be used to determine molecular orientation and alignments^18,19^. It can also be used in imaging methods^20^. As a fluorescent molecule, the anisotropy of NADH can be measured to determine its degree of rotational freedom, as well as that of any structure it binds to, such as complex I in the mitochondria. This technique can therefore be used to determine how well aligned the cristae of the mitochondria are and it can also provide valuable insights into mitochondrial biology and potential quantum properties of NADH, i.e. possible similarities between NADH and chlorophyll which has been well studied in quantum biology.

In this paper, we report that NADH in live cells is critically ordered in mitochondria. As reported by Picard et al.^11^ this might not only be the first fluorescence-based observation in live cells of inter-mitochondrial cristae alignment, but potentially, it may provide information about the intra-mitochondrial alignment that controls energy production in the ETC. We also speculate that this could, with the emerging evidence of quantum effects in biology, and the very large fields created by mitochondria, hint at the existence of field-based homeostasis and organisation. Furthermore a link between ordered structure and quantum properties have been previously made for other biological structures such as microtubules and DNA^31,32^. It is suggested that the ordered nature of tryptophans in microtubules allow for super radiance whereby an incoming photon has the same probability of exciting not just one tryptophan but the ordered collection. This increases the efficiency of photon absorption and emission.

## Materials and methods

All chemicals and reagents were of the highest purity (> 98%), unless otherwise stated, were purchased from ThermoFisher Scientific (GibocoTOM) or Merck-Sigma-Aldrich. All reagents were dissolved in a three-stage deionised water with 18 MΩ resistivity obtained from a reverse osmosis (RO) water treatment system. Analytical grade organic solvents were used where needed. The experiment was divided into two stages, with the first stage focusing on solution phase anisotropy of NADH, and the second stage on measuring the anisotropy in isolated mitochondria and cells.

### NADH solution studies

NADH stock solutions of 1 mM were freshly prepared prior to every experiment. It was found that solutions that were older than 24 h from initial preparation gave inconsistent results during excited state measurements. The stock solution was further diluted to the required varying concentrations. If other solvents such as ethanol, methanol, etc. were used, the NADH powder was directly dissolved and diluted in those solvents. A final working concentration of 33 μM was used unless otherwise stated. This concentration was found to give an excellent signal at short multiphoton acquisition times for solution and imaging studies. This allowed comparison with the cell studies where the concentration of NADH is suggested to be as low as 10 μM and high as 160 μM. For longer term studies, for use over a 24 h period, samples were wrapped in aluminium foil at room temperature or at 4 °C. For solution studies on the microscope stage, a 10 μl aliquot of the solution was placed on a coverslip and onto the microscope stage. For larger volumes, 3 ml of solution was added to a cuvette and the experiments performed in a dark experimental box.

### Glycerol dilutions for anisotropy studies

Immediately prior to the varying viscosity experiments, a mixture of deionised water and glycerol was prepared (0–95% ratio). Careful mixing of these glycerol:water solutions is critical to obtain a good NADH solution. For solution phase viscosity measurements, 10 μl was placed onto a microscope coverslip and time-correlated single photon counting (TCSPC) performed (see below). The same technique was used to obtain both the steady state anisotropy and excited state properties of NADH. Measurements were also performed in 3 ml cuvettes outside the microscope using a custom-built anisotropy apparatus. This configuration was necessary due to the effect of reducing the anisotropy value by high numerical aperture (> 0.5) microscope objectives.

### mNeonGreen fluorescent protein effect on NADH

A stock solution of mNeonGreen in 140 μM aliquots (a kind gift from Prof Dan Mulvihil and Dr Tara Eastwood, University of Kent) was stored in − 20 °C until needed. An aliquot was then thawed on the day of the experiments and diluted to a concentration of 25 μM in deionised water, unless otherwise specified. This protein was found to be unstable at room temperature for more than 24 h. mNeonGreen protein was found to form crystals easier than the other fluorescent proteins, hence the reason for their use here.

### mNeonGreen crystals for instrument characterisation

Purified mNeonGreen in 1 × phosphate buffered saline (PBS) was exchanged into 20 mM 4-(2-hydroxyethyl)-1-piperazineethanesulfonic acid (HEPES) pH 7.0 using a PD Spintrap™ G-25 column (Cytiva). Crystals were grown at 20 °C using the vapour diffusion method from a solution containing 12 mg/ml mNeonGreen mixed with precipitant containing 25% medium molecular weight polyethylene glycol (PEG) smear and 0.1 M HEPES pH 7.5. Diamond shaped crystals appeared after 24 h and grew to final size after a week.

### MCF7 cell culturing

All reagents used for cell culturing were purchased from Thermofisher Scientific (Gibco™). Michigan Cancer Foundation-7 (MCF7) cells were originally obtained from The European Collection of Authenticated Cell Culture and were stored in liquid nitrogen until cultured. The cells were cultured in a 5% CO_2_ in air, 37 °C humidified incubator using phenol red-free low glucose (1 mg/mL) Dulbecco’s Modified Eagle’s Medium (DMEM) supplemented with 10% foetal bovine serum (FBS), 2 mM glutamine and 1% penicillin/streptomycin and passaged every 2 days. For imaging experiments, cells were seeded at a density of 1 × 10^5^ cells/ml per well in 4-well glass bottom chamber slides (Ibidi, Germany) or a 3 ml glass bottom dish (Ibidi, Germany) and incubated for 24 h before imaging.

### Mitochondrial isolation

Functional mitochondria were isolated from MCF7 cells using a Mitochondrial Isolation Kit for cultured cells from Abcam (Abcam, UK, ab110170). Briefly, approximately 4 × 10^7 ^cells were harvested and centrifuged at 1000 G for 5 min. The resultant pellet was resuspended in Hanks' Balanced Salt Solution (HBSS) and centrifuged at 1000 G for 5 min. Supernatant was discarded and the pellet was frozen overnight at − 80 °C. The pellet was then defrosted and resuspended in Abcam Reagent A to a concentration of 5 mg/mL total cellular protein. The mixture was incubated on ice for 10 min, and then homogenised with 30 strokes of pestle B in a Dounce homogeniser. The homogenate was centrifuged at 1000 G for 10 min at 4 °C and the supernatant removed and set aside. The pellet was then resuspended in Abcam Reagent B, homogenised with 30 strokes of pestle B in a Dounce homogeniser, and centrifuged again at 1000 G for 10 min at 4 °C. Supernatants from both centrifugations were combined and centrifuged for a final time at 12,000 G for 15 min at 4 °C. The resultant supernatant was discarded, and the pellet containing isolated mitochondria was resuspended in 500 µL (Abcam Reagent C). To confirm mitochondrial viability and to differentiate mitochondria from background artefacts, Mitotracker green, 35 nM (ThermoFisher Scientific) was added to a separate aliquot of thawed isolated mitochondria 30 min before imaging following the manufacturer’s instructions for cell imaging (488 nm excitation/ 520 nm emission). Microscopy slides of the isolated mitochondria were prepared by combining 10 μl of isolated mitochondria with 20 μl of low melting point agarose (1%) between two coverslips. Isolated mitochondria were imaged using a × 60 objective (NA 1.2) on a Nikon Ti-E microscope and confocal system, with one photon excitation for the Mitotracker or multiphoton for the NADH (see below).

### Solution phase transient absorption (TA) spectroscopy of NADH

Time-resolved UV-NIR absorption is a pump–probe technique that uses a pump pulse to initiate a reaction which in this study is the photoionisation of NADH. A second light pulse, a probe that is time-delayed, is used to interrogate the reaction dynamics. The formation and depletion of species is determined by measuring the absorbance changes as a function of time. We used the time resolved multiple probe set up at the Central Laser Facility of the Rutherford Appleton Laboratory with the apparatus described previously^33^. We have used a silicon line array to detect transmitted probe beam. The experimental set up employed two synchronised ultrafast lasers, one with a repetition rate of 1 kHz for excitation and another (probe) at 100 kHz. The two ultrashort laser pulses were arranged so that for every pump pulse, a hundred probe spectra are collected and pump–probe time delays up to 1 ms are accessible. Briefly, excitation (or pump) was performed at 360 nm with ~ 200 fs pulses of 270 nJ at 1 kHz focused to a spot size of 150 μm FWHM (full width at half maximum). Supercontinuum probe pulses are generated on a sapphire window by focusing a fraction of 1030 nm fundamental. The probed beam was at a magic angle polarisation in the spectral range of 500–900 nm with 30 micron FWHM. The liquid sample was flowed through the beam to minimise sample degradation and ensure a fresh spot was irradiated by each pump pulse. A sample with an optical density (OD) of 0.3 at 365 nm was freshly made and used.

### Solution phase excited state lifetime and fluorescence microscopy

**Figure 1:**
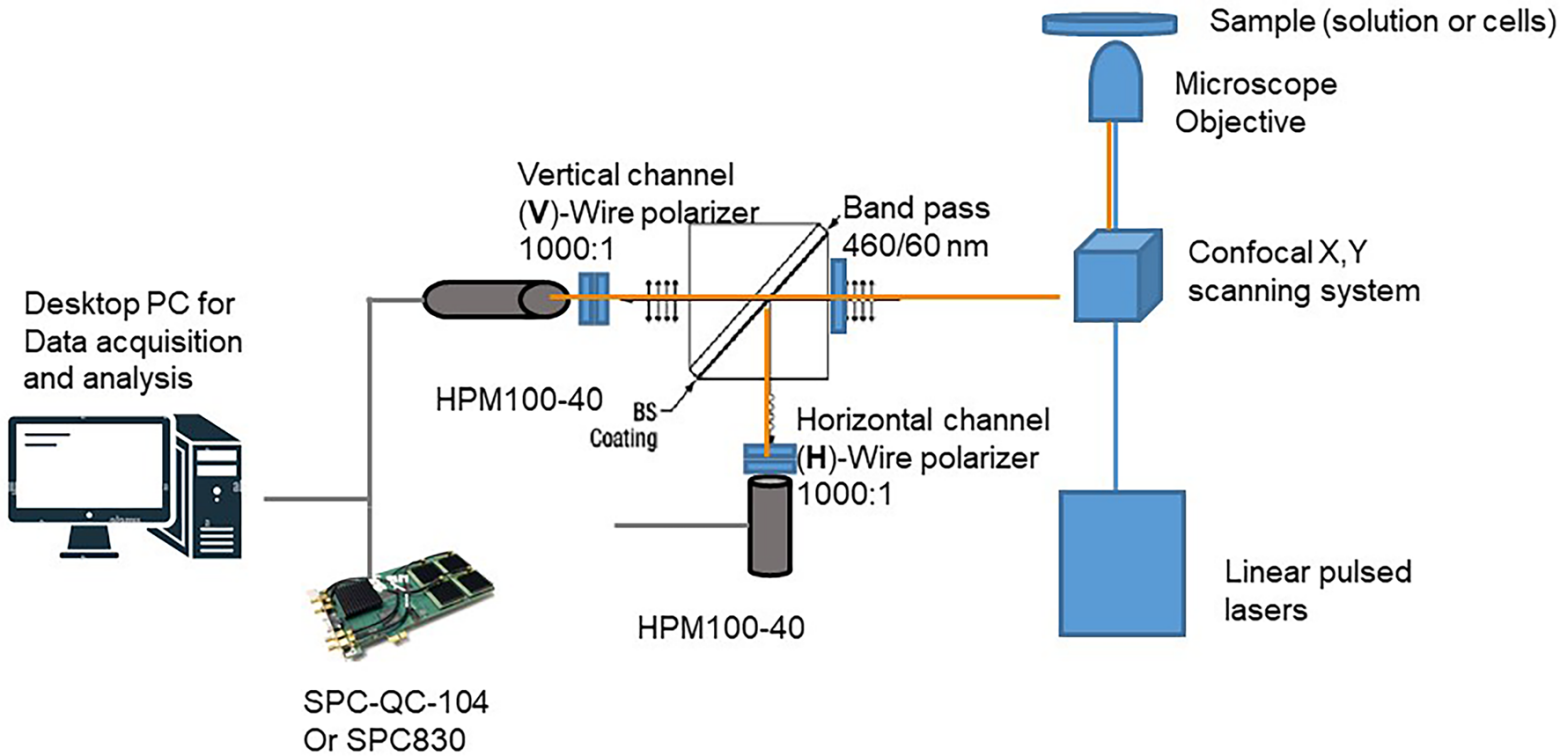
Schematic of the experimental setup. Two HPM100-40 hybrid photon counting detectors are matched for near identical operation. Directly in front of these are wire polarisers to give a 1000:1 polarisation selection as VV or VH. Extinction ratio of BS, Reflects S Polarisation (horizontal), TP:TS > 1000:1, Rs:Rp ~ 100:1.

A modified Nikon C2 confocal scan-head was used for the anisotropy imaging. This was also configured for either one photon or two-photon excitation, Fig. 1.

For the one photon excitation, a Nikon confocal scanning unit attached to a Nikon TE2000-U inverted microscope was used. This was equipped with a Super K Extreme NKT-SC 470–2000 nm super continuum laser (NKT Photonics, purchased though Photonic Solutions, UK) (variable repetition rate; 60 ps pulse width, linearly polarised light). The laser repetition rate was reduced to 39 MHz. Microscopy images were acquired with a set of objectives × 10 (NA 0.5), × 20 (NA 0.75), × 40 (NA 0.9), × 60 (NA 1.2) water immersion objective. The excitation wavelengths were 365 nm for NADH and 491 nm for mNeonGreen. Vertical and horizontal image data were acquired using a Becker and Hickl TSCPC SPC830 or SPC-QC (purchased from Becker and Hickl, GmBH) running the TCSPC software v 9.77. A PicoQuant MultiHarp 150 TCSPC module running a SymPhoTime 64 software was also used to acquire further anisotropy images and analysis. We always aimed for count rates of 1 × 10^4^ to 5 × 10^5^ photons per second. Our fluorescence lifetime imaging microscopy (FLIM) set up can be operated as a two-channel system via a router (HRT-41, Becker and Hickl for the SPC830 or the SPC-QC TCSPC module with no router required). The filters used for NADH and mNeonGreen were 460/60 and 525/39 nm (Thorlabs, Germany) respectively. The fluorescence emissions were detected as free space launch rather than the standard fibre coupled light collection to avoid depolarising the emission before detection.

Multiphoton excitation was performed using a modified Nikon EC2 confocal scan-head^34^, similar to the one-photon excitation but with the fibre excitation optics removed. Fluorescence images were acquired using 730 nm from a coherent Mira 900F (pumped by Verdi 532 nm continuous wave (CW) laser, Coherent Laser, UK), 76 MHz, 180 fs). The laser light passed through a cube polarising beam splitter (Thorlabs CCM1-PBS251/M) and a half-wave plate tuned to a wavelength suitable for two-photon excitation 400–800 nm. The vertically polarised and focused laser light was then scanned across the sample using the Nikon EC2 confocal. Fluorescence intensities of the polarisation components vertical (parallel) and horizontal (perpendicular) to that of the laser and laboratory space were collected simultaneously at two detectors containing a wire grid polariser or by rotating a sheet polariser (1000:1, Thorlabs WP25M-VIS). This was to account for any anomalies and differences in the detectors. Fluorescence signals were non-descan (bypassing the confocal optics) and detected by Becker and Hickl hybrid PMTs (HPM100-40) as shown in Fig. 1. For system characterisation and solution studies, we acquired four intensity (I) data points taken per experiment using polarisers, one located between the excitation source and the sample and the second between the sample and the emission detector: I_VV_(λ), I_VH_(λ), I_HH_(λ), and I_HV_(λ), where subscript V refers to the vertical and H to the horizontal polarization for excitation and emission specific wavelengths (λ), respectively. The use of non-de-scan with multiphoton allows higher photon collection compared to the second pass descan confocal mode. This reduces the image acquisition time and therefore essential for reducing the possible movements of mitochondria in live cells; such movement with time was unavoidable. The collections of two channels simultaneously also avoids other errors that might result from collecting vertical and horizontal images sequentially. The data from the fluorescence intensities for the two channels were analysed on a pixel-by-pixel basis. The maximum anisotropy or theoretical limits of one-photon and two-photon excitation is 0.40 and 0.57 respectively at a zero degree angle between excitation and emission. This is due to the difference in photo-selection angles that equates to roughly r_o_ of 2/5 and 4/7 respectively^35^. It is therefore advantageous to utilise a multiphoton excitation if possible for anisotropy studies.

The images were collected at 256 × 256 pixels with each pixel containing a full decay profile. This data was analysed using Becker and Hickl SPCImage V7.1 software. For the data analysis, only a photon count of 1000 or more than 100 counts in the first signal channel was used for the decay statistics and the anisotropy measurements. In the SPCImage software, it is necessary to discard pixels with poor photon signal-to-noise ratio (less than 100 counts) by adjusting the threshold range appropriately. Where photon counts are below the limits set above, a binning factor of 2 or 3 was applied to increase the photon count for the analysis. Emission decay kinetics are analysed as mono- or multi-exponential functions.

For solution-phase studies the number of photons detected in each polarisation channel were analysed using Eq. (1). Where the G-factor has been determined using a solution of known anisotropy close to zero^36^.

1 $$Anisotropy{ }\left( r \right) = { }\frac{{I_{VV} - GI_{VH} }}{{I_{VV} + {\text{G}}2I_{VH} }}$$

Anisotropy determination, where r = anisotropy, I_VV_ = photons in vertical polarisation, I_VH_ = photons in horizontal polarisation and G = the Grating or instrumentation factor for light path difference). The value 2 in G2I_VH_ is due to total intensity of any given emitter being equal to the total of the intensities (I_T_) along the three axes: I_T_ = Ix + Iy + Iz. Hence Iz becomes Iz = Ivv + 2I_VH_.

For isolated mitochondria and live cell studies, the use of high numerical aperture (NA) microscope objective (0.75 or higher) is required and so a new parameter, lens depolarisation (L) is generally required to calculate anisotropy accurately (Eq. 2)^36^. The high NA objective for the polarised excitation and light collection introduces a degree of polarisation loss which needs to be accounted for. Determination of the L value is prone to large errors. Our studies shows that our data may increase by as much as 20% but not consistently. We have therefore chosen not to apply this value to our data set.

2 $$Anisotropy \left( r \right) = \frac{{I_{VV} - I_{VH} }}{{\left( {2 - 3L_{1} } \right)I_{VV} + \left( {1 - 3L_{2} } \right)I_{VH} }}$$

Anisotropy equation for high NA objectives, where r = anisotropy, I_VV_ = photons in vertical polarization, I_VH_ = photons in horizontal polarization and L_1_ and L_2_ are the lens depolarisation values.

Prior to imaging studies, it was necessary to make sure the excitation and emission light polarisation orientations are well established and known. The multiple mirrors in the excitation beam path (including the x, y galvanometer) means the initial polarisation output from the laser (light source) is unlikely to be the same at the microscope stage. This therefore needs to be established with reference to the imaging setup. This is different to the solution phase setup which is relatively straight forward using polarizing beam splitters, a waveplate and plate polarizer for vertical and horizontal polarizations.

In this work, we have employed single crystals of mNeonGreen to aid in establishing the correct polarisation at the microscope stage. Crystals grown (as above) were initially imaged with a white light stereo microscope.

Polarisation imaging was performed using either one-photon excitation from a super continuum NKT laser or via multiphoton excitation from a coherent laser, Mira 900 (see above).

## Results

### Electron generation from UVA photo excitation of NADH

We performed a transient absorption experiment between 500 and 800 nm following a pump wavelength of 360 nm of aqueous solution of NADH and observed an absorption in the probe wavelength that is indicative of the presence of hydrated electrons, Fig. S2. Our current data showed a linear or one-photon photoexcitation process, in agreement with other reports and confirming those of Boldridge et al.^28^. It is interesting to note that such a significant electron generation into the cell energy landscape has not been fully exploited in the literature, i.e. the relationship of UVA light absorption of NADH (and by extension mitochondria) and energy production. Although we did not investigate any polarisation characteristics of the electron ejection, it is expected this will be the case also. Considering the significant levels of UVA from the sun that reaches the earth, this may have significant consequences for life.

### Excited state characteristics of NADH in solution

NADH in solution is prone to degradation, in particular oxidation. We performed excited state lifetime measurements following one and two photon excitation at 365 nm and 730 nm respectively with emission maximum at 460 nm at 18 °C. Freshly prepared sample consistently gave a NADH fluorescence lifetime of 244.9 ps ± 6.68 and 874.1 ps ± 25.8 for measurements taken at the vertical polarisation. For the horizontal polarisation, NADH had lifetime values of 473.0 ps ± 19 and 1191.3 ps ± 107.209. This is similar to that previously reported by others^37^. Considering the significant difference between the vertical (parallel) and horizontal (perpendicular) emission lifetime of NADH in water, we investigated the effects of other environments around the NADH, in particular those that are likely to affect the dynamics of functional groups. NADH dissolved in ethanol gave excited state lifetime values of 326.0 ps ± 5 and 1137 ps ± 34 for the vertical polarisation and 619 ps ± 29 and 1333 ps ± 68 for the horizontal polarisation. This shows an increase in lifetime when NADH is dissolved in ethanol that is significantly different to when dissolved in water. It is worth noting that NADH dissolved in D_2_O gave lifetimes of 262 ps ± 5.099 and 898.667 ps ± 6.799 for the vertical polarisation and 628.333 ps ± 13.474 and 1174.333 ps ± 64.075 that is not too dissimilar to H_2_O, as shown in Fig. S3 and Table 1.

**Table 1:** Summary of fluorescence lifetime parameters of NADH in different solutions.

Solvent	VV lifetime 1	VV lifetime 2	VH lifetime 1	VH lifetime 2
Water	244.9 ± 6.7	874.1 ± 25.8	473.1 ± 18.5	1191.3 ± 107.2
Ethanol	326.3 ± 4.5	1136.7 ± 34.0	619 ± 29.5	1333 ± 68.0
D_2_O	263.5 ± 4.4	921.5 ± 22.2	623.3 ± 14.5	1217.8 ± 72.2
+ Protein (mNeonGreen)	3093.5 ± 75.7		3789.7 ± 137.9	

An example of the power of anisotropy to report different environments, larger and smaller changes of NADH. Values are given as mean/ SD from a minimum of three experiments.

NADH excited state lifetime and anisotropy values significantly increase when bound to other biomolecules such as proteins. We determined values of 2452.7 ± 260.1 (τ_1_) and 3630.3 ± 152.2 ps (τ_2_) when NADH was complexed with mNeonGreen in solution. This was performed to allow NADH comparison in mitochondria (isolated and in live cells).

It was noticed that extremely high purity NADH (98%) maintained stability over time so that solution lifetime did not change over 24 h at room temperature. However, the fluorescence intensity of NADH did increase as the solutions aged. The reason for this increase is unknown.

### Solution phase characterization of NADH in glycerol:water mixtures

**Figure 2:**
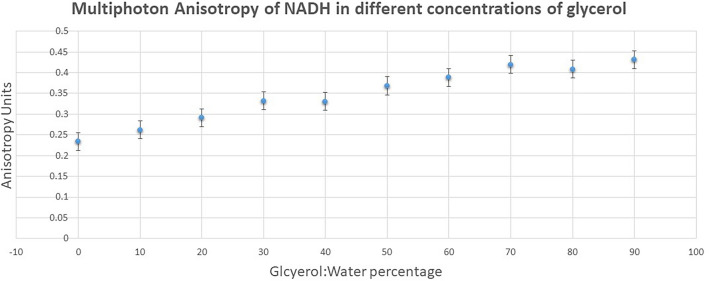
Anisotropy of 33 μM NADH in different concentrations of glycerol/water mixtures following two-photon excitation at 730 nm and emission through BG39 filter (330–660 nm). Raw data not adjusted for the high NA microscope objective (also see Fig. S5).

Figure 2 shows the anisotropy of NADH in different concentrations of glycerol/water mixtures following excitation with two-photon 730 nm, with emission at 460/60 nm. The change in the ratio of glycerol:water mixture did not affect the excitation and emission maxima. The anisotropy of NADH increased as the environment became restrictive with decreasing water percentage. The anisotropy of NADH was 0.24 ± 0.02 in water alone. Whilst at 90% glycerol concentration, the anisotropy rose to 0.43 ± 0.02 for a vertical excitation and emission following multiphoton excitation. The reason for the higher than usual NADH in water alone compared to other small molecules such as fluorescein and coumarin is unknown. However, it is clear that simple NADH molecules in solution may be adopting favourable dipole orientation with the excitation electric field vector. It was also observed that the decay characteristics of NADH required a single exponential decay fitting at the high glycerol concentration. This was determined at glycerol concentrations more than 80% with χ^2^ between 0.9 and 1.3—the measure of how best the calculated decay fitted to the decay data. The reason for the high anisotropy in water alone is unknown. The current data represent a raw data output and not adjusted for the high NA objective effect (the L-value, see Eq. 2 and Fig. S4).

### Solution phase characterization of 7-hydroxycoumarin carboxylic acid and fluorescence in glycerol:water mixtures

As a comparison, we determined the anisotropy characteristics of 7-hydroxy coumarin carboxylic acid (7-OH-CCA) and fluorescein in glycerol/water solutions. Our measured excited state and lifetime values are similar to those previously published with anisotropy values of 0.01 ± 0.021 and 0.07 ± 0.02, respectively in water alone and rising to 0.38 in 90% glycerol environment for both probes, Fig. S4.

### Anisotropy characterisation of mNeonGreen crystals

**Figure 3:**
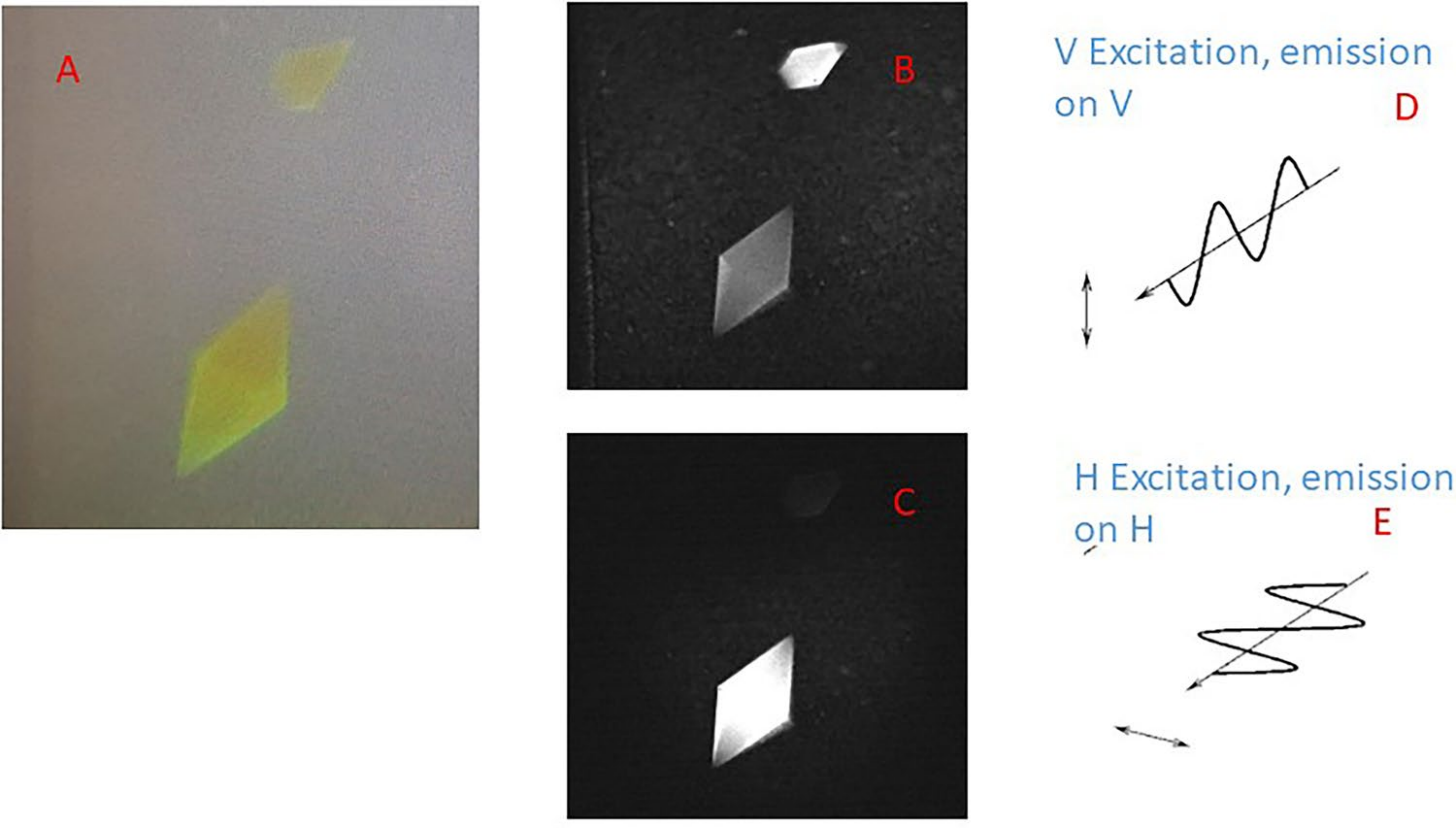
Characterising the imaging setup using of mNeonGreen crystal for polarisation calibration, (**A**) is a white light image of two crystals in the field of view, (**B**) is a confocal fluorescence image following excitation with parallel (vertical) excitation and emission, (**C**) is horizontal excitation and emission, (**D**, **E**) represent the excitation electric fields for the vertical and horizontal excitations. Fluorescence image, 488 nm excitation, 515/40 nm emission, 10 × objective, 370 μm FoV.

Once the solution phase studies were established, we turned to fully characterise our anisotropy imaging setup. Single crystals of mNeonGreen were used to ensure the correct polarisation at the microscope stage with respect to the laboratory vertical and horizontal axis. Figure 3 shows a white light transmission image of two crystals with orthogonal orientation to each other. Following excitation with the vertical and horizontal polarisation with the similar orientation emission, the molecular alignments of the molecules within the crystals with respect to the excitation becomes apparent. We confirm the orientations further by rotating the excitation polarisation using waveplates (λ/2). We were able to confirm clear orientation preferences of the crystals so that their intensities reduced by a factor of 3 when the excitation or emission polarisation was set perpendicular to the crystal axis. The waveplate allowed rotation of the excitation light at the microscope state whilst wire polarisers (with 1000:1 extinction ratio) were used to select the emission. This routine allowed us to confirm the polarisation at the microscope stage ready for the mitochondria and cell studies.

### Anisotropy characterisation of isolated mitochondria using the natural NADH fluorescence

**Figure 4:**
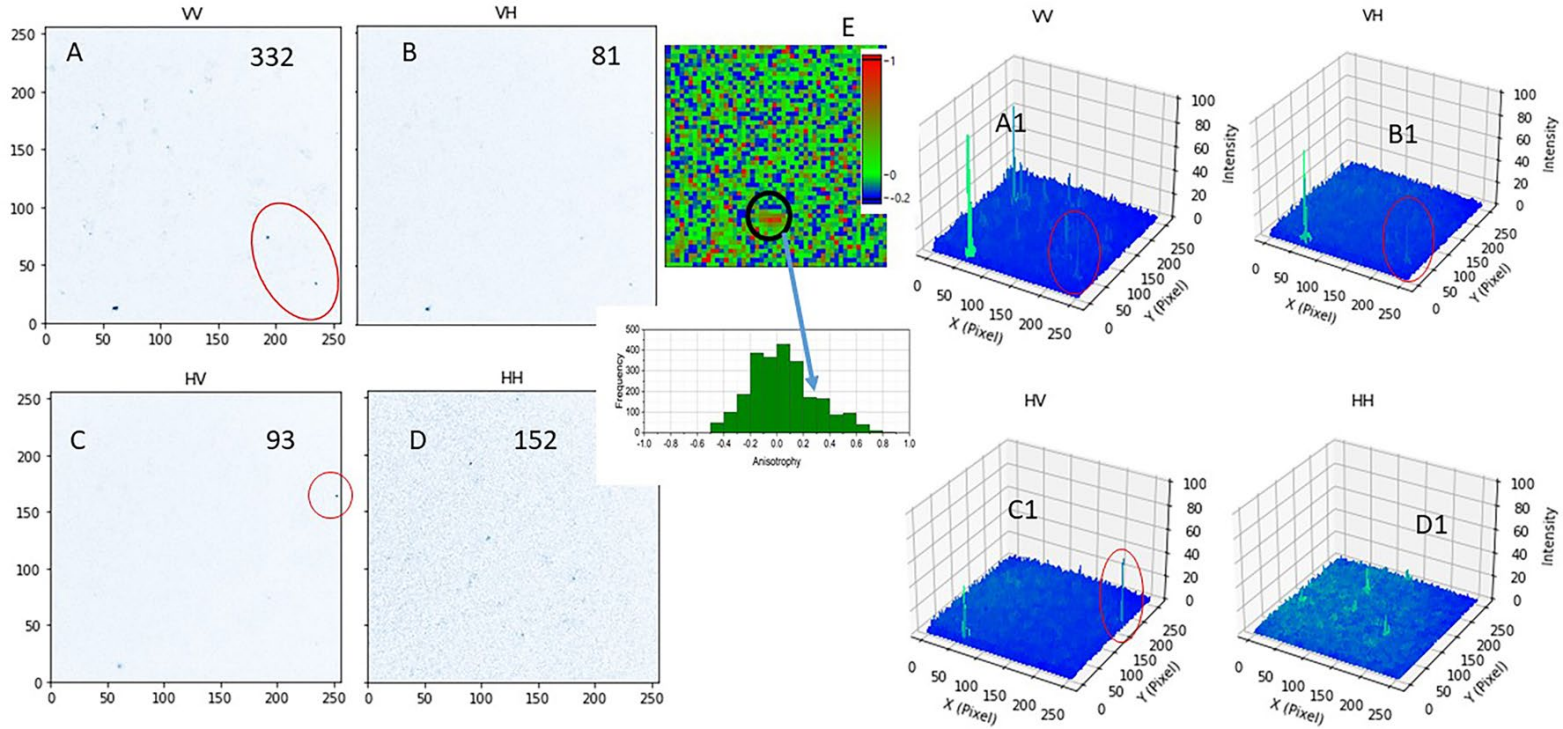
Two-photon fluorescence images of natural NADH in Isolated Mitochondria from MCF7 cells. Time resolved decay profiles were acquired from circled regions to confirm NADH, shown in Fig. S3. Representative images from 3 repeat experiments. Red circles also represent polarisation specific emissions. (**A**) is vertical excitation and emission, (**B**) is vertical excitation, horizontal emission, (**C**) is horizontal excitation, vertical emission, (**D**) is horizontal excitation and emission. A1–D1 are the equivalent count profile plots where difference in intensities are reflected, (**E**) is an anisotropy map of and pixel distributions. Arrow shows higher anisotropy of mitochondrion compared to large empty space of zero anisotropy. FoV 50 μm.

Once the correct excitation and emission polarisation at the microscope stage was established, we proceeded to image isolated mitochondria from mammalian cells. Figure 4 shows steady state fluorescence anisotropy images of isolated mitochondria following multiphoton excitation (at 730 nm) with either vertical or horizontal excitation and subsequent emission (through a BG39 filter) in the same (vertical) or perpendicular (horizontal) orientations. We observe from the intensity images significant difference when different polarisations were applied. The presence of (isolated) mitochondria was confirmed by observing a fluorescence lifetime that is similar to the NADH in solution and cells. The identified features in the confocal and multiphoton field of view showed two component excited state lifetimes similar to (protein) bound NADH of around 350 ps (τ_1_) and 2 ns (τ_2_) (Fig. S5). When the isolated mitochondria were also labelled with Mitotracker green and following one-photon excitation at 491 nm, (to confirm presence of mitochondria), we again observed these same (punctate) features in the field of view as those from multiphoton excitation. Images were sequentially taken, first with 491 nm then with the multiphoton excitation. The punctates showed an anisotropy values of 0.35 ± 0.07. We have also observed values as high as 0.5, close to the theoretical limit. The anisotropy value is similar to those we obtained for example fluorescein and NADH in 80% glycerol. This is significantly larger than that expected if the NADH within these structures are free floating and not tethered. Our current data using anisotropy imaging indicate that the NADH being excited within the isolated mitochondria are likely highly aligned, due to the polarisations change.

### MCF7 and HeLa cell anisotropy imaging

**Figure 5:**
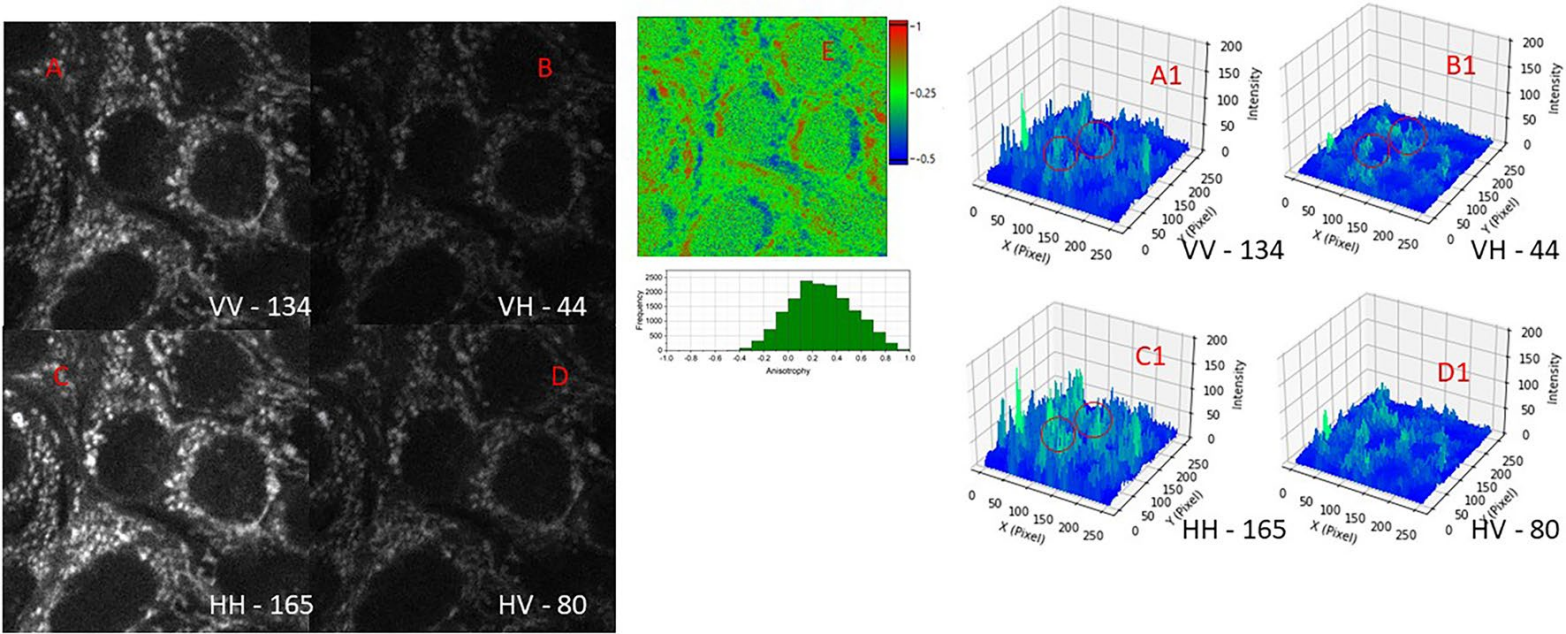
Two-photon fluorescence images of natural NADH in mitochondria from MCF7 cells. Time resolved decay profiles confirm NADH. Representative images from 3 repeat experiments. (**A**) is vertical excitation and vertical emission, (**B**) is vertical excitation, horizontal emission, (**C**) is horizontal excitation, vertical emission, (**D**) is horizontal excitation and horizontal emission. A1–D1 are the equivalent count profile plots where difference in intensities are reflected. Red circles also represent polarisation specific emissions, (**E**) is an anisotropy map of and pixel distributions. FoV 50 μm.

**Figure 6:**
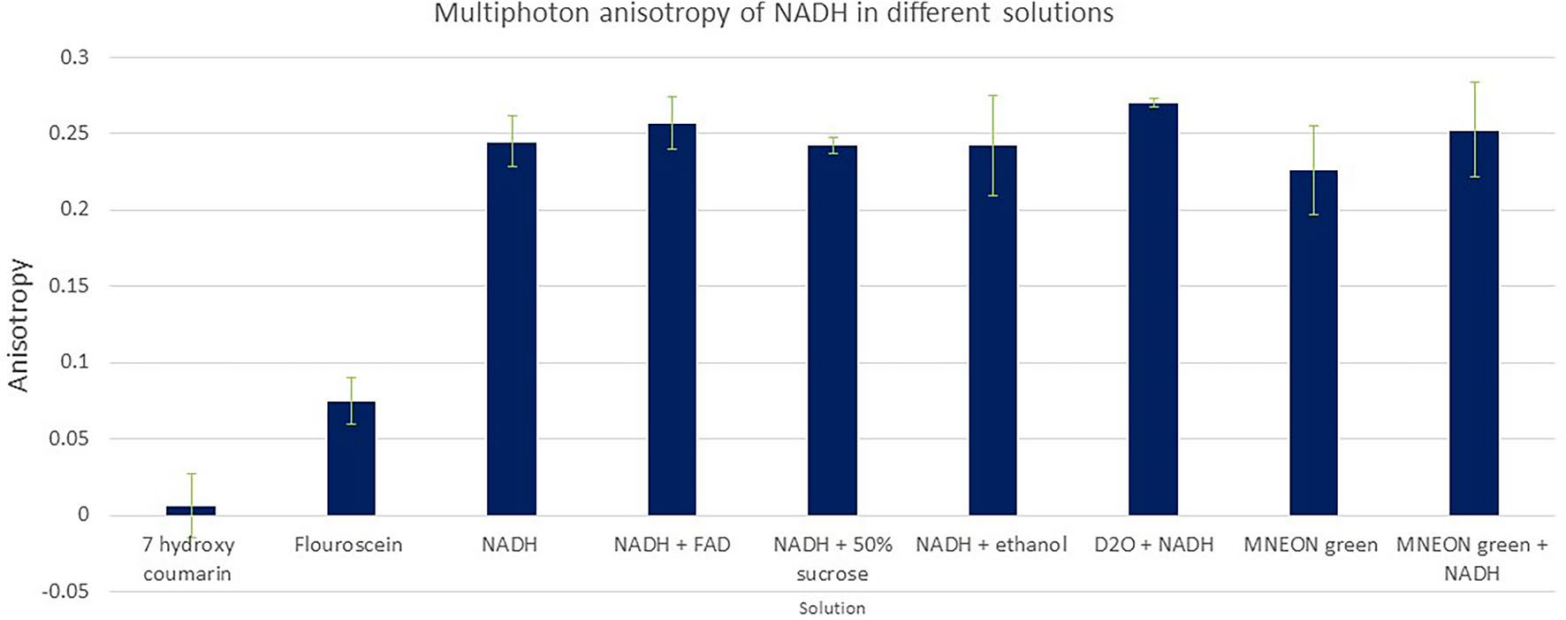
Anisotropy of NADH in different solution environments mixtures following two-photon excitation at 730 nm and emission through BG39 filter (330–660 nm). 7-OH-CCA and fluorescein are used as representative small molecules for comparison. NADH anisotropy is significantly influenced by its environment and can be used as a good reporter of cellular and mitochondria environment.

Figures 5, S6F and S6E shows the fluorescence images of MCF7 and HeLa cells following excitation with both vertically and horizontally polarised light as done for the isolated mitochondria. Emission was collected in both the parallel (vertical) and perpendicular orientations (horizontal). Images were acquired from the same area of the cell with only the polarisations being altered. The intensity values (red circles) show that the intensity of an area of the cell is higher for similar polarisation and emission whilst cross-polarisation (VH and HV) shows significantly less intensities. Analysis of these images using Eq. (1) gave anisotropy values between 0.3 and 0.5, similar to those we obtained for example fluorescein and NADH in more than 80% glycerol. Again, this shows a much hindered environment with little Brownian motion. Interestingly we imaged autofluorescence of plant fibres in tissue paper (as a control) and found no anisotropy (value < 0.1 using Eq. 1) even though these are dried solid and fixed samples. We conclude that the fluorescent molecules in these tissue samples do not show any clear alignment compared to NADH molecules in the mitochondria organelles. See Figs. 6 and S6, overall summary. These data therefore also shows that the NADH is highly likely aligned in the mitochondria within cells, and by extension, in the cristae too. The anisotropy values of the NADH within these mitochondria clearly show that they are aligned to a large extent as evidenced by the polarisation preference orientations.

## Discussion

Alignment and directionality is seen as important and as critical factors in quantum biology. Examples include the alignment of chloroplast containing chlorophyll and retina light sensitive cells. In this study, we used fluorescence anisotropy to investigate the alignment of NADH in intact cells and isolated mitochondria. NADH, which exhibits autofluorescence, is predominantly found in mitochondria cristae, and thus constitutes an effective approach for studying their shape and function. The underlying hypothesis is that mitochondria are highly morpho-dynamic and show clear evidence of structural alignment that relates to metabolic function and cell shape. This is mirrored by their internal cristae morphology, and that as they create their own electric fields and photons, it is possible, given the importance of quantum mechanics and thermodynamics in biology, that life may be utilising a field-based “electrodynamic” form of homeostasis. The origins of this could relate to the fact that life is defined by the movement of charge, and thus the creation of electric fields, both dynamic and static, and the fact that it is full of chromophoric molecules with key quantum properties that are essential for it to function.

In relation to the technique, multiphoton excitation allows improved anisotropy measurements over one-photon processes^35,38^. Our solution phase studies complement earlier time resolved and anisotropy literature reports that have been applied to understanding the biophysical properties of NADH^37^. The key here is that photon absorption and a possible electron ejection may have a significant quantum mechanical process. How much fundamental quantum effects played a role in the origins of life will almost always be impossible to determine, but authors have speculated on it^39–41^. A related molecule to NADH found in the mitochondria, flavin adenine dinucleotide, (FAD) has been reported to respond significantly in a magnetic field. Flavin-based autofluorescence in native, untreated HeLa cells was found to be magnetic field sensitive, due to the formation and electron spin–selective recombination of spin-correlated radical pairs^4^.

### NADH anisotropy may be indicative of a level of bioenergetic organisation

Our preliminary data on NADH anisotropy does suggest a possible level of bioenergetic organisation that has been highlighted but has been difficult to prove^42,43^. It is important to note that redox signalling is fundamental to every aspect of life and certainly occurs in the mitochondria, coupled to the tricarboxylic acid cycle (TCA cycle), is highly compartmentalised^44,45^. Indeed, even within a mitochondrion, different parts of the cristae can be doing different things^46^. Thus, control of spin states, ROS and bioenergetics, as well as field-based effects on voltage-gated ion channels, could all be integrated with changing fields as a form of feedback.

We suggest that mitochondria could be dependent on multiple quantum effects to function, not previously thought possible due to the “warm and wet nature” of life, where fields play an important role in the feedback mechanism. The fact that NADH appears to show directionality, which although it could have a pure physical “classical” basis (alignment with membranes), is also worth a consideration. In particular since the charge density across the inner mitochondrial membrane is suggested to be around 30 million volts per meter^2^, and if spin in electron movement is playing a role, this may well affect the electron transfer from, for example, NADH to FeS complexes in complex 1. The energy is thought to be transferred by way of an electrostatic wave against the proton motive force (PMF) to pump a proton across; conformational shifts in the protein are thus key, as a kind of charge dependent piston action^47^. Practically, NADH passes its electrons to flavin mononucleotide (FMN), and then on through a series of FeS proteins and thence to ubiquinone; this system can go into reverse and reduce NADH. Critically, it is thought that quantum tunnelling may well be key in how this may work^48,49^. Altogether, at a local level in the cristae, there is likely to be very intense fields that control function. It could be argued that, for instance, the field effect on the initial FMN-NADH binding could be relevant. This becomes all the more poignant because membranes can be viewed as capacitors, due to the large voltages across them, meaning they experienced electrostriction^50^. It has been long known that the ATPase controls membrane bending^51^, and that it thus determines the local PMF at cristae rims^52^. Taken together, mitochondrion cristae directionality and induced electron flow becomes an important structure to consider.

### Tentatively linking NADH and quantum biology

The link between NADH and quantum biology has not been previously proposed; we suggest this link because not only is NADH ubiquitous in all life indicating its ancient origin, but also because it is highly sensitive to UV, which has been pivotal in determining the course of life on earth and NADH is intimately involved in the movement of charge. Here, we attempt to relate the initial NADH photoionisation to the process of photosynthesis where a photon from sunlight is absorbed by the thylakoid, ejects an electron from the chlorophyll, producing an exciton in the process. Absorption of UV by a cell can fundamentally change its metabolism, depending on the intensity, duration, and the metabolic state of the cell. This process may be best described by quantum mechanics (in the initial event), but can result in a well described biological biphasic response call hormesis, which is tightly linked to redox^8^. We believe that the high yield of mono-photonic electron generation (~ 50%) is significant. For example, even if a small fraction of these electrons produced undergo a quantum mechanical event, the outcome would be a profound quantum biology process, and could well be influenced by factors such as field direction and strength.

It is now clear that electromagnetic fields, such as from rotating magnets, can profoundly influence mitochondrial function, for instance, as an approach to kill cancer cells^53^, or at low intensities (10 µT) and low frequency (1–8 Hz) to induce mitophagy and rejuvenate mitochondrial function^54^. Other data also suggest that prolonged EMF can enhance cardiac and osteogenic mitochondrial function—the proposed mechanism involving a mild production of ROS and possibly complex I involvement^55,56^. Interestingly, earlier work showed that magnetic fields could affect the enzyme activity of horseradish catalysed oxidation of NADH. The mechanism proposed involved the first step and was related to the single electron transfer and the formation of the NADH^+^ radical cation^57^. The possible inference here is that the first step of the proton-coupled electron transfer (PCET) at complex 1 involves the close proximity of NADH and FMN aromatic rings; the electrons are then passed down a series of FeS centres to quinone; the conformational structure is critical to prevent potential production of ROS^58^. If components of the ETC are highly organised and ordered, then EMF could potentially affect spin states and thus PCET, with the NADH/FMN interaction being a key target.

### Solution phase NADH anisotropy

The oxidized form, NAD+, is non-fluorescent and is generally not considered as significant in cell imaging. It is thought that the functional group in NADH responsible for the fluorescence is the reduced nicotinamide ring (Fig. S7). It is worth mentioning that the excited state lifetime of NADH in aqueous solution has two components, τ_1_ ~ 250 ps and τ_2_ ~ 500 ps with pre-exponential factors of ~ 70% and ~ 30% respectively, giving an average of around 400 ps. Despite previous numerous studies, the exact origin of these two lifetime components are still unknown. This fluorescence, mostly from the nicotinamide is also partially quenched by collisions with molecules in the vicinity or stacking with the adenine moiety (Fig. S7).

Using the fluorescence emission of NADH, we observed a high steady state anisotropy value for NADH in PBS, ethanol and methanol in the absence of any other molecule following multiphoton excitation. The origin of NADH’s anisotropy is very much debated in the literature and is still unknown. Our determined anisotropy value of 0.2 in aqueous solution rising to 0.44 following two photon excitation is slightly lower to that previously published (0.5–0.55)^52^. Our data has not been corrected using the L-factor in Eq. (2) (Fig. S4). The effect of this would increase our initial anisotropy value close to that of ~ 0.5. The initial anisotropy (r_0_) is purely a property of the fluorophore excitation and emission dipoles, (a relationship between the angle of absorption and emission). In general, highly symmetric molecules exhibit some polarisation effect due to the selective electric field influence from the excitation photons (as mentioned above). A theoretical maximum for r_0_ values are 0.4 and 0.57 for one-photon and two-photon events respectively. It is therefore advantageous to perform anisotropy measurements using a multiphoton process. Furthermore, the molecular weight may also have an effect on the polarisation correlation time, i.e. the rotational mobility time (ϕ) associated with a depolarising process. This is generally much shorter than the fluorescent lifetime (τ). The rotational rate of the molecule as a whole in solution of the chromophoric region (together with size and shape) is dependent on the viscous drag imposed by the solvent. Considering a simple small molecule dissolved in a low viscosity solution, it is expected that the rate of rotational diffusion is typically much faster than the rate of emission so that the fluorescence is depolarised resulting in the anisotropy being close to zero. This is in agreement with our observations for 7-hydroxycoumarine and fluorescein but not NADH in water, methanol and ethanol. The size difference leading to the high anisotropy of NADH and other similarly sized molecules is a subject of debate in the literature. Moreover, how much the shape of NADH- stack of the adenine and nicotinamide ring plays in the high initial natural anisotropy observed is also unknown^59^.

Ultrafast transient anisotropy measurements have been performed to determine the polarisation relaxation sensitivity in NADH in the excited state using polarisation-modulation pump-probe spectroscopy^60^. It was suggested that dynamics of anisotropy is dependent on the solvent, such as ethanol concentration, solution polarity and viscosity. In our D_2_O and ethanol–water studies, we observe both increases in the excited state lifetime and anisotropy on NADH. This might be due to the fact that ethanol is less polar than water and therefore to a lesser extent perturbs the NADH wave function leading to faster relaxation and stronger influence on the NADH nuclear configuration^61,62^. These are all conditions that are present and critical in mitochondria in cells. We have not considered the isotropic transient signal following pump-probe studies for NADH in the excited state which is beyond the scope of this work. This would require performing quantum mechanical chemical calculations of vibronic and electronic wave functions in the initial electron energy states of solution phase NADH to allow solving for the vibrational density energy matrix.

### Imaging mitochondrial inner structure alignment

The ultrastructure of mitochondria, in particular the cristae, inner and outer membrane have been imaged at tens of nanometre resolution following labelling with external fluorescent probes (for the former) or using transmission electron microscope for the latter two^63,64^. These previous studies have shown both ultrastructure and bulk mitochondria structure alignment. It has been suggested that this alignment could control function, and with evidence of alignment between mitochondria at IMJs (Fig. S1b), this could be a way to improve efficiency^12^. However, no studies so far have used the natural fluorescence of NADH contained within the mitochondria to determine possible alignment. As yet, there are no super-resolution methods that uses the NADH for such imaging at the sub-one hundred-nanometre resolution. Here, our use of anisotropy spectroscopy and imaging partially addresses possible NADH alignment in mitochondria in a simple way.

Mitochondrial structure is known to undergo numerous alterations and adaptations under varying cellular conditions. For example, mitochondria undergo fission, fusion, loop or donut structure formation, which are related to stresses, such as starvation—donuts, for instance, can maintain mitochondrial membrane potential^65^. There is also the “chicken and egg” question, do mitochondria change shape in response to shifts in metabolism, or does their shape change to alter metabolism; it is likely both, with evidence indicating mitochondrial morphology can control fatty acid utilisation^66^. Fusion and fission is also key in their calcium signalling function, for instance, signals can be transmitted quickly when they are in reticular network, but halted when they become separate^67^. Considering the involvement of NADH in the energy generation of mitochondria, it may very well play a role during the structural changes as well as the correlation relationship between form and function. The conformation of NADH has been shown to be dependent on metabolic state investigated by fluorescence anisotropy. The NADH molecule is also reported to have some sensitivity to environmental changes such as viscosity (a change of 0.5 mPa*s) and hypoxia^24^. We have previously speculated that the balance between coherence and decoherence, in effect, the boundary between classical and quantum effects, could be a key way biology adapts to the ever changing environment—the “quantum mitochondrion”^16^. The ability of NADH to generate electrons efficiently (Q_Ye_, 0.44) versus that of fluorescence (Q_F_, 0.02), is perhaps an example of this adaptability.

## Conclusion

NADH is a truly ancient molecule and was probably pivotal in the abiotic to biotic transition of life. It is pivotal in redox, and its association with other key molecules, such as the flavins and FeS compounds within complex protein architectures, suggest a need for a high degree of directional order. Although preliminary, our data does highlight important and interesting properties of NADH that might help understand the possible quantum basis of mitochondria and life. Biology is perhaps at a crossroads, with the acceptance that it is fundamentally electrical, and now with data emerging that even its shape could well be related to a morphogenetic field, with ion channels playing a key role. The emerging anisotropic properties of NADH in mitochondria and cells is a step towards shape and chirality-induced selectivity. Finally, and significantly, light-dissipating properties of NADH where the photon-electron generation is highly controlled requires future studies. It is tempting to speculate that the ability of NADH to absorb a photon and eject an electron, could have played a role in early life. Our future studies will aim to develop methods to directly image NADH at super resolved limits, below 100 nm as well as determine the directionality of photoelectrons from NADH.

### Supplementary Information

Supplementary Information.

## Data Availability

The datasets used and/or analysed during the current study available from the corresponding author on reasonable request. These are stored on UKRI-STFC server for 10 years.
