# Supplementary material for: The use of NADH anisotropy to investigate mitochondrial cristae alignment
Source: Sci Rep. 2024 Mar 12;14:5980. doi: 10.1038/s41598-024-55780-5 (PMC10933486; doi:10.1038/s41598-024-55780-5)

**The Use of NADH Anisotropy to Investigate Mitochondrial Cristae Alignment**

Holly. E. Smith^1^, Alasdair M. Mackenzie^1^, Chloe Seddon^2, 3^, Rhys Mould^4^, Ifi Kalampouka^4^, Partha Malakar^1^, Sarah R. Needham^1^, Konstantinos Beis^2,3^, Jimmy Bell^4^, Alistair Nunn^4^, Stanley W. Botchway^1*^

^1^UKRI, STFC, Central Laser Facility, Rutherford Appleton Laboratory OX11 0QX, UK

^2^School of Life Sciences, Imperial College, London, UK

^3^Rutherford Appleton Laboratory, Research Complex at Harwell; Didcot, Oxfordshire OX11 0FA, UK

^4^Department of Life Sciences, Research Centre for Optimal Health, University of Westminster, London, W1W 6UW, UK

*Stan.botchway@stfc.ac.uk

**Supplementary Figures**


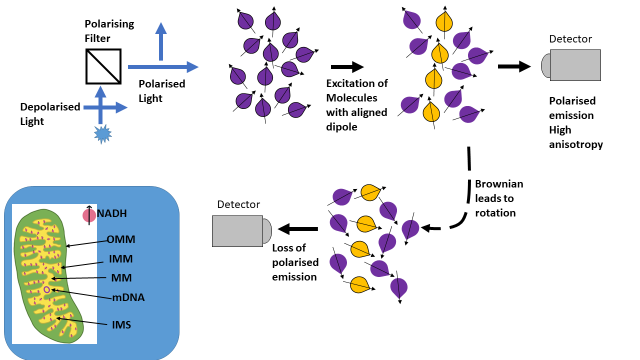


Figure S1- Schematic of photo selection excitation of molecules (e.g. NADH) by polarised light. Bottom left, sketch of mitochondrion and possible alignment of NADH molecules along cristae with enhanced photon selection excitation and emission. **OMM** -Outer Mitochondrial Membrane, **mDNA**- Mitochondrial DNA, **IMM**- Inner Mitochondrial Membrane, **MM**- Mitochondria Matrix , **IMS**- Inter-membrane Space

Figure S2- Signal in transient absorption as function of the pump-probe beam intensity of NADH aerated solution (water). **A**) expected electron absorption. **B**) Signal as a function of the pump beam average energy (nJ).

B

A


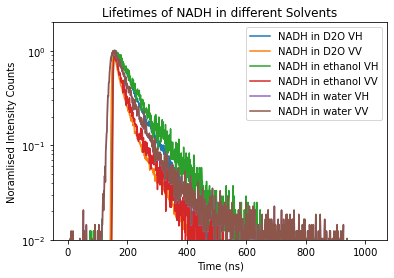


Figure S3- Effects of solvated environment of free NADH following one-photon excitation.

Figure S4. Effect of microscope objective numerical aperture (NA) on anisotropy Multiphoton NA values vs NADH anisotropy. Using a solution of NADH in water alone black bar, NADH in 50% glycerol:water and fluorescein in water a lone for comparison (blue).


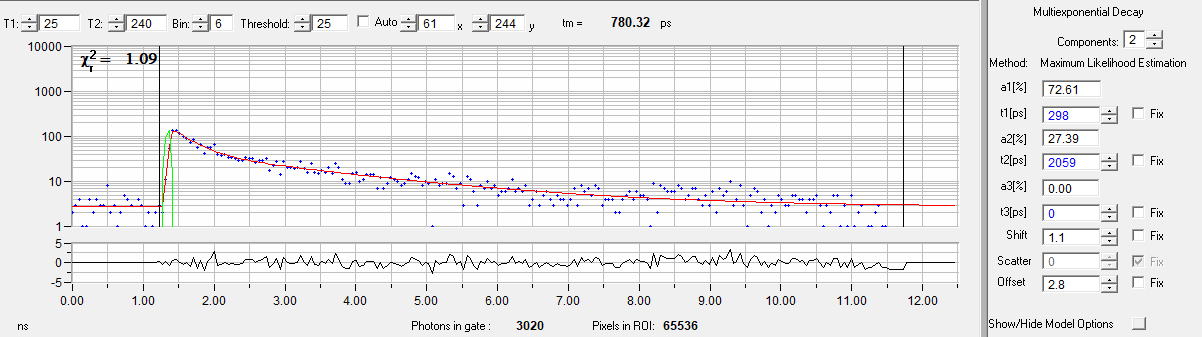

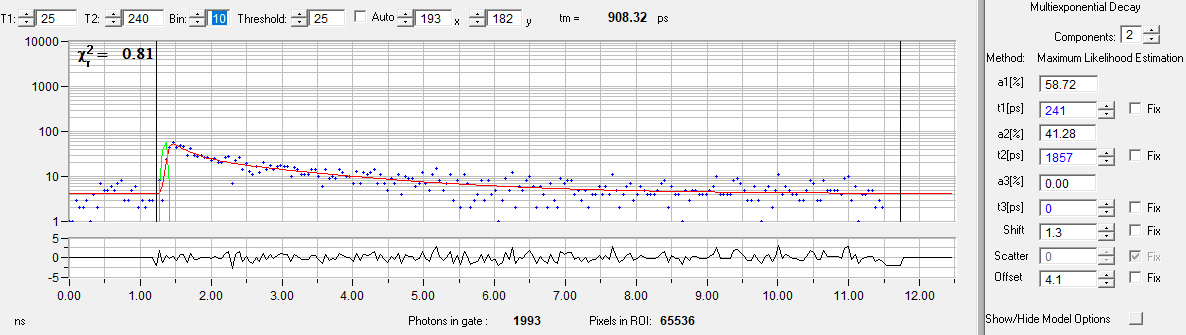


Figure S5, A typical decay from isolated mitochondria (from figure 4) following multiphoton excitation at 730 nm and emission through BG39 (330 – 650 nm). Vertical (top) and horizontal (bottom) emissions with decay analysis.


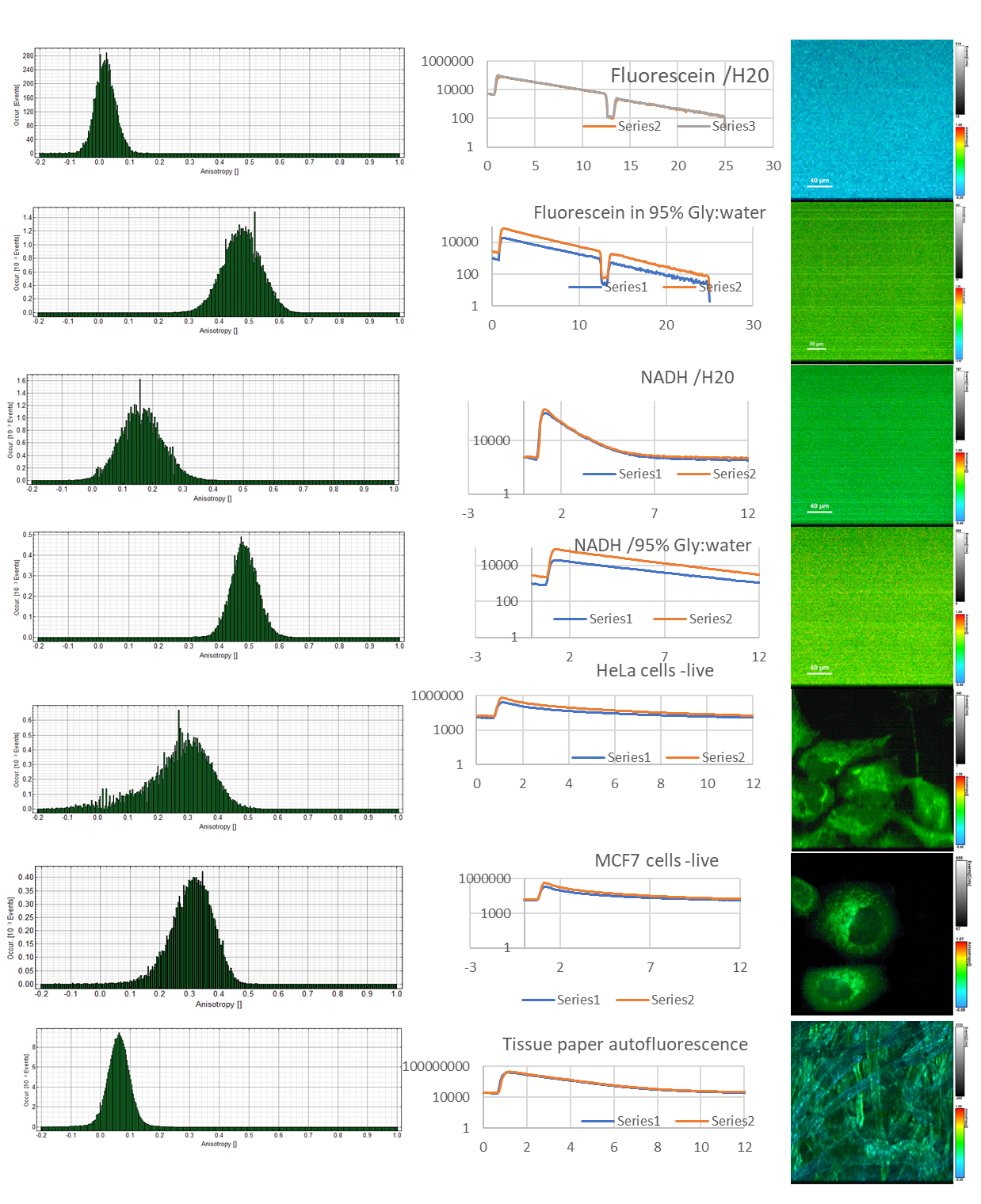


**F**

**A**

**B**

**C**

**D**

**G**

**E**

**A**

**B**

**C**

**D**

**E**

**F**

**G**

Figure S6- Two-photon (730 nm) fluorescence anisotropy (r_o_) images of compounds in solution and cell. Representative images from at least 3 repeat experiments. Row **A**, distribution of anisotropy across the image of fluorescein water solution, middle decay trace of VV and VH intensities and left, anisotropy colour map image of the solution. Row **B**, distribution of anisotropy across the image of fluorescein 90% glycerol:water solution, middle decay trace of VV and VH intensities and left, anisotropy colour map image of the solution. Row **C**, distribution of anisotropy across the image of NADH water solution, middle decay trace of VV and VH intensities and left, anisotropy colour map image of the solution. Row **D**, distribution of anisotropy across the image of NADH 90% glycerol:water solution, middle decay trace of VV and VH intensities and left, anisotropy colour map image of the solution. Row **E**, distribution of anisotropy across the image of live HeLa cell at room temperature, middle decay trace of VV and VH intensities and left, anisotropy colour map image of the solution. Row **F**, distribution of anisotropy across the image of live MCF7 cell at room temperature, middle decay trace of VV and VH intensities and left, anisotropy colour map image of the solution. Row **G**, distribution of anisotropy across the image of tissue paper autofluorescence, middle decay trace of VV and VH intensities and left, anisotropy colour map image of the solution. The FoV 80 um

Figure S7, Structure of NADH


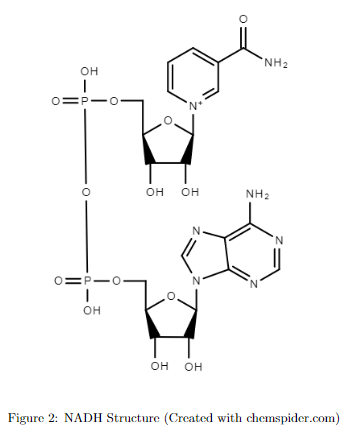

Supplement: Supplementary file 1 — Supplementary Information. [file 41598_2024_55780_MOESM1_ESM.docx]
